# Gene Expression-Guided Drug Repurposing in Oncology: Insights from Antiretroviral Agents in Prostate and Bladder Cancer

**DOI:** 10.3390/genes17020184

**Published:** 2026-01-31

**Authors:** Mariana Pereira, Nuno Vale

**Affiliations:** 1 PerMed Research Group, RISE-Health, Faculty of Medicine, University of Porto, Alameda Professor Hernâni Monteiro, 4200-319 Porto, Portugal; mariana.m.pereira2097@gmail.com; 2 ICBAS—School of Medicine and Biomedical Sciences, University of Porto, Rua de Jorge Viterbo Ferreira, 228, 4050-313 Porto, Portugal; 3 RISE-Health, Department of Community Medicine, Health Information and Decision (MEDCIDS), Faculty of Medicine, University of Porto, Rua Doutor Plácido da Costa, 4200-450 Porto, Portugal; 4 Laboratory of Personalized Medicine, Department of Community Medicine, Health Information and Decision (MEDCIDS), Faculty of Medicine, University of Porto, Rua Doutor Plácido da Costa, 4200-450 Porto, Portugal

**Keywords:** transcriptomic signatures, Connectivity Map, LINCS L1000, functional genomics, precision oncology, biomarker stratification, prostate cancer, bladder cancer, antiretroviral repurposing

## Abstract

**Background/Objectives**: Gene expression-guided drug repurposing has emerged as a strategy to identify new therapy opportunities by associating disease transcriptional signatures with drug-induced gene expression profiles. This is relevant for prostate and bladder cancers, which have high molecular heterogeneity and therapy resistance limits for their standard treatment regimens. Antiretrovirals have been of great interest as repurposed candidates for these cancers due to their various effects on cancer cell pathways. The objective of this review is to assess the principles, applications, and challenges of this approach, with emphasis on antiretrovirals. **Methods**: This review summarizes published literature on gene expression-based drug repurposing methodologies, including signature reversion, pathway level analysis, and validation studies. Studies applying these concepts to prostate and bladder cancer were analyzed, and evidence of antiretroviral repurposing for cancer therapy was assessed based on transcriptomic alterations, pathway perturbation, and preclinical outcomes. **Results**: Transcriptomic-driven studies identified several drug candidates capable of modulating gene expression associated with therapy resistance, tumor progression, and cell stress responses. The anticancer effects of antiretrovirals were shown to be related to cell cycle arrest, apoptosis, metabolic alterations, and proteostasis. Nonetheless, transcriptomic responses are highly context-dependent and can be influenced by tumor subtype and experiment and treatment conditions. Off-target effects can also complicate mechanism interpretation. **Conclusions**: Gene expression–guided drug repurposing enables the systematic prioritization of clinically actionable candidates by matching disease and drug transcriptional signatures, but successful translation will require the integration of other omics results, careful model selection, and the development of clinically relevant biomarkers to support mechanism-informed repurposing. Translation will depend on subtype-aware signature matching, integration with complementary omics, and biomarker-backed validation to support precision deployment.

## 1. Introduction

The field of oncology remains marred by numerous challenges globally, both due to high mortality rates and substantial financial burdens. Different methods for discovering new therapies are being searched for every day, and one that has been of interest lately is drug repurposing [1]. This strategy allows for the identification of new indications for previously established drugs, which accelerates therapy development, while at the same reducing costs and risks, since these drugs are already shown to be safe [2]. Prostate and bladder cancer remain some of the most common types of cancer existing worldwide and still present challenges for the treatment of some subgroups, making these cancers prime candidates for drug repurposing [3,4]. One drug group that has been more studied for cancer repurposing is antiretrovirals. Coincidentally, one of the first antivirals developed for HIV treatment, zidovudine, was initially designed as an antineoplastic capable of altering cell cycle genes and causing decreased proliferation in ovarian cancer cells [5,6,7]. Several other antiviral drugs have been shown to decrease cancer cell viability and impair other oncogenic pathways over the years [8,9,10,11,12,13].

For drug repurposing, the use of gene-expression signatures is being favored over specific molecular targets, as they are more commonly used. Comparing the transcriptomic signature of diseases to the gene expression changes induced by drugs can be used to select drug repurposing candidates that can reverse or mitigate alterations in gene expression caused by the disease [14]. The objective of this review is to understand how changes in genetic expression, pathway modulation, and transcriptomic–phenotypic relationships can be used to uncover anticancer mechanisms and to reposition drugs originally developed for non-oncological diseases, with a particular focus on antiretroviral agents and their preclinical evidence of repurposing in prostate and bladder cancer.

In a precision oncology context, transcriptome-guided repurposing is most impactful when it moves beyond candidate ranking to patient stratification, linking disease/drug signatures to clinically deployable biomarkers and resistance mechanisms. While several reviews have addressed repurposing in oncology and the specific example of antiretrovirals, those tend to focus on phenotypic outcomes and target-based mechanisms. This review aims to center gene expression-guided repurposing, emphasizing how disease and drug-induced transcriptomic signatures can be used in repurposing. It also highlights the relevance of including the subtype-specific transcriptomic profiles of both prostate and bladder cancer for signature reversal at a subtype and pathway level. By integrating all of these parameters, this review will show how repurposing can be refined into a context and clinically dependent strategy instead of the usual one-size-fits-all approach.

## 2. Gene Expression-Guided Repurposing

The idea behind gene expression-guided repurposing is that, for each disease and drug, there is a transcriptional signature associated with them, and that good drug candidates for repurposing should either mimic the effects of protective genes or reverse disease-related expression patterns [15]. Typically, in oncology, drug signatures are derived from cells, either immortalized cell lines or primary cells, that are exposed to the chosen drug for defined time and concentration points [14]. Meanwhile, disease signatures are usually obtained by tissue comparisons between tumoral and normal tissues or between sensitive and resistant tumor tissues [16]. To achieve this, several resources can be used.

**Figure 1:**
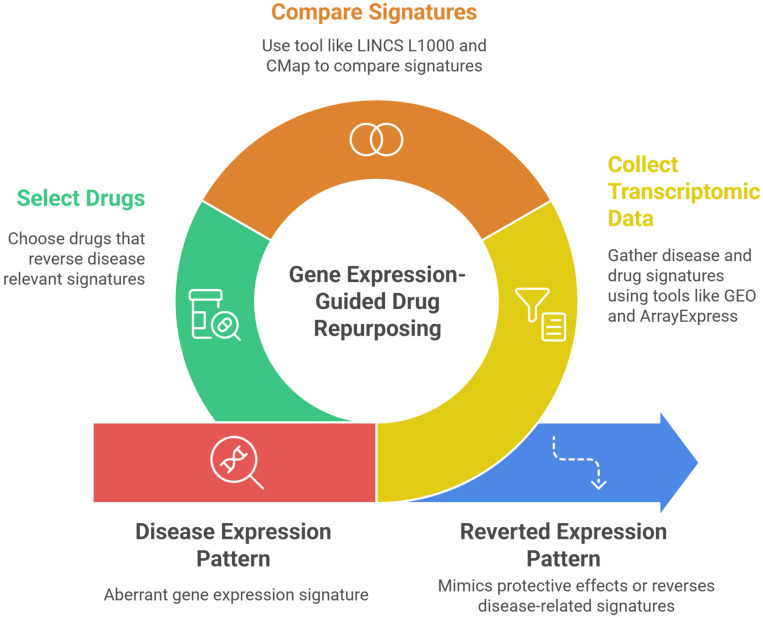
Workflow for gene expression-guided drug repurposing.

The Connectivity Map and the successor LINCS L1000 are programs where thousands of compounds and their respective induced expression profiles can be found. The LINCS L1000 has over 1 million gene expression profiles that are derived from many human cell responses to drugs and is able to discover new mechanisms of action. It also permits the matching of drug and disease signatures via similarity and enrichment scoring [17]. There also exist several public repositories that contain disease transcriptomes from either microarrays or RNA-seq, which can be mined to find differentially expressed genes or gene signatures. Some examples include ArrayExpress and Gene Expression Omnibus (GEO) [18]. Typically, the workflow for this type of research would initiate with collecting the transcriptomic data (the signatures) and using tools like LINCS L1000 to compare signatures, selecting the drugs that cause a signature reversion of the disease. This would be either downregulating some genes or upregulating suppressed genes, and it is usually ascertained by the connectivity score [19]. A representation of this workflow can be seen in Figure 1.

This type of workflow has proven to be fruitful. A study by Fisher [20] used several methods of signature reversion to compare the signatures of cancers, like glioblastoma, liver, lung, and pancreatic carcinomas, with the ones from several repurposed candidate drugs, focusing on those with more promising results in the literature [20]. The results showed that pamidronate, a bisphosphonate used for hypercalcemia [21], and nimodipine, a calcium channel blocker [22], were predicted for glioblastoma, and in vitro testing performed by the same team validated the in silico results, showing inhibited tumor growth [20]. Similarly, another study used this approach to evaluate candidates for colorectal cancer repurposing and found 12 FDA-approved drugs that reverse the disease signature, 4 of which (tezacaftor, fenticonazole, bempedoic acid, and famciclovir) were tested in vitro. Two colorectal cancer cell lines (HTC-116 and HT-29) were selected, and MTT and RT-PCR assays were performed, confirming that these drugs modulate genes shown in the in silico predictions. As an example, we can cite the decrease in *SOX4* and modulated *SFN* expression caused by tezacaftor. This work demonstrated that signature reversal predictions are translatable into biologically relevant changes [23].

However, it is not always the case that gene reversion is necessary or optimal. For example, Koudjis [24] showed that the predictive power of signature reversal was more associated with a global anti-proliferative effect than with specific disease pathways [24]. Signature reversal can also be too simplistic, and it is essential to include network-focused strategies, since diseases are a combination of interconnected genes and regulatory networks. Sometimes, it may be important for a repurposed drug to alter key genes or central hub genes rather than invert the transcriptome of a disease [25]. Gene-level signatures can also be varied due to aspects like cell-type heterogeneity, so focusing on a more pathway and network-level analysis can help to reduce noise while maintaining all of the rich information and increasing the robustness and understanding of complex gene expression patterns [26]. Pathway enrichment approaches in which drug and disease-induced expression changes are mapped onto pathways or regulatory networks offer results that are easier to interpret biologically than single gene lists [27]. Some resources that can be used for pathway analysis are the Kyoto Encyclopedia of Genes and Genomes (KEGG) database [28] and the Reactome Pathway Database [29], which provide pathways and group genes by function and process. These types of repurposing strategies can also go into clustering diseases according to functional and mechanistic similarities. This allows for the identification of drug candidates that interfere with dysregulated processes that are common to the diseases. Combined disease–drug networks can reveal modules of diseases with similar perturbations and drugs that can act on those diseases [30]. Machine learning tools can also be used to integrate other types of information, such as molecular targets and chemical drug structures, to further improve results [31].

## 3. Antiretroviral Agents as Anticancer Candidates

A direct relation between antiretroviral therapy (ART) and anticancer effect in people living with HIV is controversial. A North American study evaluated 22 cohorts of patients between 1996 and 2014 and the differences in the risk of cancer developing between early and late ART start [32]. The study found that early ART treatment led to a decrease in cumulative cancer incidence, with a decrease in risk of 30%. Kaposi’s sarcoma and non-Hodgkin lymphoma, two AIDS-defining cancers [33], had the highest decrease, with 68% and 42%, respectively, and in a 15-year follow-up, there was a lower cumulative incidence of virus-related non-AIDS-defining cancers. Despite these findings, no decrease in incidence was detected for non-AIDS-defining cancers that are not of viral origin, which led to the conclusion that ART on cancer reduction is likely due to immune reconstitution and better protection against oncogenic viruses [32]. Another meta-analysis of more than 47 thousand HIV-positive patients in North America, Europe, and Australia showed a high decrease in both Kaposi’s sarcoma and non-Hodgkin’s lymphoma, while showing no changes for any of the other cancer types [34]. Despite these findings, studies on this topic have the limitation of all being observational and not incorporating any mechanistic investigations, such as the use of biomarkers or analysis of tumor tissue [35].

Aside from these findings, there is already some direct evidence that antiretrovirals could be useful in cancer treatment. Some mechanisms of the anticancer effect of antiretrovirals that make them relevant include the following: cell cycle arrest, possibly related to decreased cyclin D1 expression [36]; endoplasmic reticulum stress induction through an increase in unfolded proteins and the expression of AMPK, which is a mTOR pathway inhibitor [37]; and autophagy, potentially related to the decreased gene expression of matrix metalloproteases, which have been associated with autophagic cell death [38,39]. Additionally, antiretrovirals have extensive descriptions of their pharmacokinetic behaviors as well as their possible interactions with other drugs, which facilitate clinical studies in oncology [40,41].

HIV Protease Inhibitors (PIs), such as ritonavir and saquinavir, are a group of antiretrovirals that specialize in blocking the AIDS virus protease essential for virus maturation [42]. They have been of great interest for repurposing. The reasoning behind this is that PIs cause side effects in patients such as insulin resistance, hyperglycemia, and dyslipidemia, which are often associated with side effects of the inhibition of the PI3K/Akt pathway [43]. Hyperactivation of this pathway occurs in most cancers and leads to cell survival, metabolic reprogramming, and metastasis, making this an important target for antineoplastic therapy [44], and PIs have been shown to inhibit Akt [43]. PIs can also inhibit P-glycoprotein [45], a major transporter, and cause cell cycle arrest [46].

Reverse transcriptase inhibitors (RTIs), including both nucleosides (NRTIs), such as abacavir and zidovudine, and non-nucleosides (NNRTIs), such as efavirenz and etravirine, impede the reverse transcription of the HIV RNA genome into DNA, effectively stopping the virus’s replication life cycle [47]. Anticancer effects of RTIs have been related to three main pathways: cell cycle arrest in the G1 phase; inhibition of the endogenous elements, LINE-1 (long interspersed nuclear elements), which are often upregulated in cancer and lead to genomic instability [48]; and DNA damage by DNA strand breakage [49].

## 4. Gene Expression-Guided Drug Repurposing in Prostate Cancer

### 4.1. Transcriptomic Strategies in Prostate Cancer

Prostate cancer has a marked interpatient and intrapatient molecular heterogeneity with multiple genomic changes between the different clinical subgroups, which make its diagnosis and treatment challenging [50,51]. Gene expression-based drug repurposing has been applied to prostate cancer with the hopes of finding drugs that can reverse the disease signatures associated with castration resistance, metastatic progression, and specific molecular targets. Irham [52] utilized a comprehensive set of genetic variants to identify risk genes for prostate cancer, which were subsequently mapped to biological pathways and potential drug targets. Using the Connectivity Map transcriptomic profiles of the PC-3 prostate cancer cell line, a drug repurposing analysis was conducted, highlighting drugs that target the androgen receptor (ESR2) and the MAP2K1/MEK pathway as top candidates. The binding strength was also confirmed with molecular docking, showing the potential of repurposing drugs, all by using transcriptomic and structural evidence [52]. Another example of gene signature reversal can be found in the investigation by Kim [53], where the gene expression signature of normal and castration-resistant prostate cancer tissue, obtained from GEO, was computed with drug-induced gene expression, obtained from LINCS, to understand the best drug candidates for this aggressive prostate cancer type [53]. Metabolic reprogramming in prostate cancer also demonstrates the relevance of the study of metabolic pathway signatures for the development of new therapeutic targets. This suggests, indirectly, that repurposed drugs that target metabolic pathways or that provoke cell cycle arrest can be of interest in this disease [54].

### 4.2. Antiretrovirals in Prostate Cancer

Ritonavir has been shown to inhibit P-gp function through the Caco-2 P-gp transporter assay and its mediated drug efflux in docetaxel-resistant prostate cancer cell lines (DU-145DOC10 and 22Rv1DOC8). The combination treatment of RIT and docetaxel led to a sensitization and resistance reversion of these cells to docetaxel and cabazitaxel [55]. Ritonavir also potentiates the proapoptotic and growth inhibitor effect of docetaxel in cancer cell lines (PC-3 and DU-145) while causing a block of CYP3A4 induction and of the DNA binding activity of nuclear factor κB (NF-κB), a major contributor to docetaxel resistance [56]. Saquinavir, another PI, caused a concentration-dependent increase in apoptosis in various prostate cancer cell lines (PC-3, DU-145, and LnCaP), possibly due to its inhibition of the 20S and 26S proteasome activity and consequent block in the activation of NF-κB. It was also demonstrated, in the same study, that sensitization of prostate cancer cells to ionizing radiation was caused by saquinavir [57]. Our research lab has also found that saquinavir causes a decrease in the cell viability of PC-3 prostate cancer cells, both alone and in combination with 5-fluorouracil [58].

On the NNRTIs side, a recent study from our research group demonstrated that efavirenz and etravirine reduce PC-3 cell viability in a time and concentration-dependent manner, with their combination impairing colony formation and consequent long-term proliferation of prostate cancer cells [59]. Although not a direct study in prostate cancer, efavirenz was flagged as having a high potential for binding and inhibiting poly-adenosine diphosphate (ADP) ribose polymerase (PARP) [60], which is involved in the DNA repair of tumor sites and is of interest in the treatment of metastatic castration-resistant prostate cancer [61].

Carlini [62] investigated the NRTI abacavir in cell lines PC-3 and LNCaP. The authors found a widespread gene expression change, which included the upregulation of LINE-1 retrotransposon transcripts as per RT-PCR results. Changes were also noted in the transcriptomic levels of genes involved in nuclear components, chromatin remodeling, protein modification, and DNA replication and repair, as obtained through the microarray assay. Possible phenotypic outcomes from these changes were obtained through pathway analysis and included cell cycle arrest in the S-phase, senescence, and impaired migration and invasion [62]. Similarly, nelfinavir (a PI) had significant alterations in next-generation RNA-sequencing profiles on PC-3 and DU-145 cells, with particular changes in transcripts related to metabolic and stress-response genes. It inhibits castration-resistant cell proliferation due to the blocking of intramembrane proteolysis [63]. Nevirapine (another NNRTI) also led to the reprogramming of androgen receptor signaling-associated and disease-progressive gene expression in hormone-refractory prostate cancer cells, downregulating the expression of genes that lead to androgen-independent phenotypes [64]. Although none of these studies had a direct signature reversal approach, they demonstrated how drug-induced gene expression profiling can result in the discovery of mechanisms for repurposing. Table 1 summarizes all the applications of antiretroviral repurposing for prostate cancer described above.

In the context of treatment for prostate cancer, these combination strategies with antiretrovirals seem to be feasible at a preclinical level but will require careful consideration. Although these drugs help to increase efficacy and combat chemoresistance, problems could arise at a systemic toxicity level due to other unknown drug–drug interactions. Clinical translation will require a study of dose optimization to ensure the successful implementation of antiretrovirals in prostate cancer, not necessarily only as replacements for established therapy, but as adjuncts to improve the standard of care.

## 5. Gene Expression-Guided Drug Repurposing in Bladder Cancer

### 5.1. Transcriptomic Strategies in Bladder Cancer

Bladder cancer has high molecular heterogeneity. The Cancer Genome Atlas Research Network has shown recurrent mutations across several urothelial carcinomas. These include genes involved with cell cycle regulation (such as *TP53* and *CDKN2A*), chromatin modification (such as *KDM6A* and *EP300*), and key tyrosine kinase and growth factor pathways. In particular, the PI3K/AKT/mTOR and the RTK/MAPK pathways have several tarobtains that have therapeutic potential [65]. Transcriptomic analysis of bladder cancer demonstrates that it can be classified into multiple molecular subgroups. A recent update on the LundTax bladder cancer classification has used microarray and RNA sequencing data of over 2500 cases to obtain the subtype-associated gene expression signatures of bladder cancer. This resulted in five classes, consisting of urothelial-like, genomically unstable, basal squamous-like, mesenchymal-like, and small-cell neuroendocrine-like [66]. There is also a general classification of bladder cancer into non-muscle invasive and muscle invasive, both of which have been shown to present several heterogeneous molecular subtypes [67,68].

The use of drug repurposing for bladder cancer is of high relevance, since, although intravesical therapies and radical surgery reduce recurrence and progression, there are still many patients who evolve high-grade or metastatic bladder cancer [69]. Cisplatin-based chemotherapy is a first-line treatment for aggressive bladder cancer [70], but the use of this antineoplastic often ends in chemoresistance and therapeutic failure [71]. Repurposing strategies using gene expression as a guide for bladder cancer have been performed before, using transcriptomic and proteomic signatures from patients with non-muscle-invasive bladder cancer. High and low-risk patient signatures were obtained and were then input into CMap to identify compounds that could reverse the disease signature of high-risk aggressive bladder cancer to that of a low-risk patient. A high-scoring compound capable of inhibiting mTOR was found (WYE-345) and validated using in vitro colony-forming and proliferation assays [72]. This study demonstrated the potential of these kinds of approaches in finding repurposed drugs for bladder cancer.

### 5.2. Antiretrovirals in Bladder Cancer

Ritonavir has been combined with the proteasome inhibitor ixazomib and has been shown in several bladder cancer cell lines (J82, 5637, and UMUC3) to decrease proliferation and cell viability in a synergistic way, with apoptosis-inducing and cell cycle-arresting effects. This was caused by an accumulation of ubiquitinated proteins and consequent endoplasmic reticulum stress and proteasome overload, leading to apoptosis, as well as by the downregulation of cyclin D1 and CDK4, which dysregulate the cell cycle. Although a transcriptomic profiling study was not performed, the molecular changes encountered can imply a shift in the stress response, cell cycle, and survival regulatory networks [73]. Similar results were found for the combination of ritonavir with nelfinavir, where bladder cancer cell death was also attributed to endoplasmic reticulum stress, as well as an inhibition of the mTOR pathway [74].

Our research group has also investigated the NNRTIs efavirenz and etravirine in a bladder cancer cell line (UMUC5) and found that both drugs had a time and concentration-dependent decrease in cell viability, with efavirenz having higher efficacy. However, 25etravirine alone, as well as the combination of these antiretrovirals, had a higher migration and colony-forming impact than efavirenz, showing that these two drugs have different anticancer effects on bladder cancer [59].

All this evidence of antiretroviral repurposing in bladder cancer is phenotypical, and, to this date, no genome-wide gene expression or transcriptomic studies have been performed. This presents a gap in the field of gene expression-guided repurposing approaches of antiretrovirals for bladder cancer that could discover the transcriptomic mechanisms of this drug class.

For drug repurposing in bladder cancer, it is also important to consider intravesical administration, where the drug is directly delivered to the bladder via a urinary catheter, which can help in surpassing the pharmacokinetic limitations of drugs that impede their clinical translation [75]. The issue in this type of administration ultimately arises from drug permeability across the bladder permeability barrier, composed of layers of several types of cells, such as basal germinal and umbrella cells [76]. The physicochemical properties of drugs influence the penetration through the urothelium, with drugs that are lipophilic and with a small molecular weight having higher permeability [77]. A positive charge also gives drugs better bladder mucosa uptake, showing that drugs used for intravesical administration also tend to comply with Lipinski’s rule of five, despite its creation for oral drug bioavailability [78,79]. Although some antiretrovirals have the characteristics for mucosa penetration, these need to be considered when trying to repurpose this drug class for bladder cancer treatment.

## 6. Challenges of Gene Expression-Guided Repurposing

One of the main challenges of gene expression-guided repurposing is in the experimental model. Immortalized human cancer cell lines are widely used in this context. However, these lack the complexity and microenvironment of tumors [80]. Components like stromal and immune cells are missing, and the heterogeneity of intratumor cells is also lacking, limiting the ability of the genetic signatures to mimic interactions that are integral to gene expression and drug responses in human cancers [81]. They also often show transcriptomic profiles different from those of primary tumor samples [82] and even diverge more the longer the cells are in the culture [83], limiting the translational relevance of the reversal signatures of drugs from cell lines to human diseases.

Prostate and bladder cancers both have biological contexts that alter drug response and gene expression. In prostate cancer, models that are androgen-sensitive rely on androgen receptor signaling for transcriptomic networks and therapies, while androgen-independent or castration-resistant models have vastly different gene expression signatures, giving these two models different drug responses [84]. Similarly, bladder cancer, as previously mentioned, has several molecular subtypes, even within non-muscle and muscle-invasive bladder cancer, with widely different gene expression and transcriptomic heterogeneity associated with different oncogenic drivers and treatment sensitivity [85,86]. Patient-derived models, like organoids and xenografts, are more useful for repurposing, since they are more faithful and better preserve the tumor heterogeneity and transcriptomic signatures of bladder and prostate cancer [87,88]. All these differences result in a drug response varying across the molecular types of both diseases, showing the importance of model choice for gene expression-guided drug repurposing.

Gene expression changes also vary over exposure time and drug concentration in drug treatment, which has been proven with systematic studies of multiple times and concentrations in different cell lines [89]. Different genes can respond at various drug doses, and dose–response curves applied to transcriptomic profiling allow us to understand the dose-dependent effects on certain cellular processes [90]. There is also a need to differentiate between early therapeutic effects and later adaptive responses, since stress and resistance-associated transcriptomic changes can arise early during treatment [91]. Integration of multi-omics can help us understand what responses are directly associated with the drug treatment and distinguish them from stress responses, separating transient from durable responses [92].

**Figure 2:**
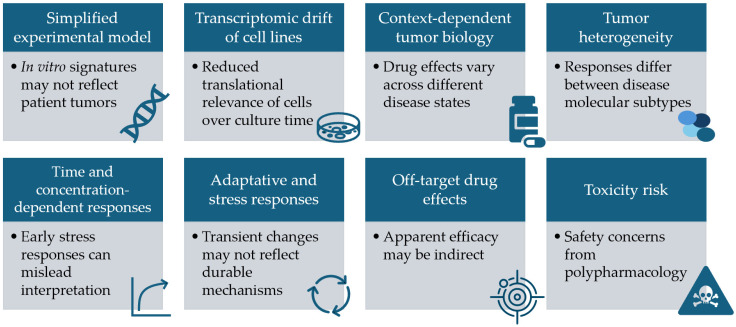
Challenges of gene expression-guided drug repurposing.

Many of the anticancer effects of antiretrovirals also seem to be derived from off-target interactions. Ritonavir, for example, is a strong CYP3A4 inhibitor, which is a common metabolizer of many drugs. Its inhibition can decrease the metabolism of other drugs and lead to a higher therapeutic effect [93]. Other antiretrovirals, like efavirenz and nevirapine, also induce and inhibit several cytochrome P450 enzymes, leading to several drug–drug interactions (DDIs) [94]. Several PIs (such as ritonavir, saquinavir, nelfinavir, and indinavir) are also known inhibitors of transporters like the breast cancer resistance protein (BCRP) and the OATP family of anion-transporting polypeptides. These PIs can then alter the efflux and uptake of drugs, which can directly interfere with their effects [95,96]. Besides these interactions with metabolizers and transporters, antiretroviral therapy has been associated with increased oxidative stress [97] and consequent mitochondrial dysfunction [98], which can lead to a positive response in cancer treatment that is not directly related to any oncogenic pathway. These off-target effects, however, are not undesirable in drug repurposing, and polypharmacology, or the use of multitarget drugs, can lead to an increase in clinical success [99]. Nonetheless, it is important to still carefully validate these effects and to establish therapeutic relevance, since, as previously stated, they can be context or tissue-specific, and there is an increased risk of toxicities [100]. Approaches such as CRISPR screening of the whole genome can help find genes whose loss affects drug sensitivity and resistance, distinguishing between primary targets and secondary adaptive changes [101]. Figure 2 summarizes the different challenges that gene expression-guided repurposing faces.

## 7. Future Directions and Translational Prospects

Future gene expression-guided repurposing efforts for prostate and bladder cancer could benefit from integrating multi-omics data instead of just transcriptomic information. Adding genomics, proteomics, and metabolomics, as well as mutational profiles and epigenetic regulation, offers further insight into the molecular complexity of cancer and improves target discovery and drug repurposing [102]. Multi-omics integration can reveal specific molecular drivers of oncogenic signaling that single-omics cannot [103] and can also help in personalizing medicine by predicting patient-specific drug responses and allowing for an optimized individual therapy regimen [104]. Machine learning has been used to perform a two-stage prediction that first clusters disease gene expression signatures and then identifies drugs that could reverse cluster-specific signatures [31]. This could be adapted to patient-specific multi-omics disease signatures and use them to stratify subgroups based on drug sensitivity, allowing for informed treatment decisions [105]. Single-cell RNA sequencing can also be incorporated. Repurposing approaches based on this, such as DrugReSC, can provide drug-by-cell matrices of how drugs can reverse the expression signals of specific tumor subpopulations of cells that are integral to cancer pathology [106].

In addition to muti-omics, incorporating computational models into pharmacokinetic/pharmacodynamic parameters and enzyme/transporter expression profiles can help with the understanding of drug exposure and response and DDIs, optimizing clinical therapy efficacy [107]. Several studies support this idea for antiretroviral repurposing, mainly using ritonavir. A physiologically based pharmacokinetic (PBPK) model incorporating drug physicochemical characteristics and enzymatic kinetic parameters was able to assess the DDI potential of ritonavir with other drugs that are substrates of CYP2D6 and CYP3A4/5 [108]. Another PBPK model of ritonavir was also validated and was able to predict the pharmacokinetics of drugs and DDIs across several CYP3A4 and P-gp substrates [109]. Even pharmacokinetic studies of combined antiretrovirals and antineoplastic drugs predicted potential DDIs between darunavir, ritonavir, etravirine, and efavirenz with erlotinib and gefitinib, allowing for the potential optimization of combination doses in drug repurposing [110]. Integrating pharmacology parameters like volume of distribution, renal elimination, metabolic clearance, and tissue penetration can help to bridge transcriptomic and in vitro findings with in vivo positive patient results [111]. The volume of distribution reflects the drug partition between plasma and tissue, and the relationship between it, tissue distribution, and plasma protein binding can impact local drug concentrations on the target [112,113]. It is not always the case that a drug with efficacy in vitro will work clinically, as was demonstrated with the study of ivermectin, an antiparasitic drug, for COVID-19 treatment. Although this drug inhibited replication of the SARS-CoV-2 virus [114], limitations for its translation were shown at a capped maximum plasma concentration that did not achieve the concentration necessary to inhibit replication, as well as a high protein binding of ivermectin and potential low tissue penetration [115]. It is also important to consider that alterations like renal impairment in patients with chronic kidney diseases alter the pharmacokinetics of drugs and can lead to inefficacy and toxicity and adjustment of the dose regimen is an important field to study, with in silico modeling and in vivo clinical trials [116]. Renal impairment also affects hepatic metabolic drug clearance pathways and drug metabolite elimination [117], further emphasizing the need to account for metabolic and renal clearance alterations when clinically translating cell results and repurposing antiretrovirals.

For the translation into clinical practice of gene expression-guided repurposing, there is a need to connect transcriptomic signatures with clinical biomarkers and outcomes. An example of this in prostate cancer is the phase II ENACT trial, where three predefined gene expression signatures (androgen receptor activity (AR-A) score, Decipher Genomic Classifier (Decipher), and the Prediction Analysis of Microarray 50 (PAM50) cell subtype signature) of biopsies from patients exposed to a drug (enzalutamide) were collected to evaluate disease progression after therapy. The study found that high Decipher scores were associated with positive reactions to treatment, and high AR-A and PAM50 scores were associated with negative biopsy incidence [118]. Another example is the STREAM phase II trial, where a correlation was formed between men who have quicker prostate cancer relapse with aggressive genotypic signatures, showing the power of transcriptomic analysis for the prognosis of this cancer [119]. The inclusion of pharmacodynamic biomarkers, such as phosphoproteins, also helps in the design of clinical protocols and can inform treatment impacts and outcomes, as these are measured post-treatment and show if the drug under study reached the target and induced the pharmacological response intended [120]. These can be obtained from mRNA expression profiles and can either be for early efficacy or target engagement [121]. The overall inclusion of biomarkers and biomarker stratification in clinical trials can help to validate predictive biomarkers in oncology and create population subgroups and tailored treatments [122].

Taken together, for the future increased success of gene expression-guided repurposing of antiretrovirals in bladder and prostate cancers, it is mandatory to include functional studies and clinical observations, linking gene signatures with response endpoints in conjunction with transcriptomic profiling. Adding pharmacokinetic models, multi-omics, and biomarker studies can further inform these kinds of investigations and lead to the discovery of personalized oncologic therapies using antiretrovirals.

## 8. Conclusions

Gene expression-guided repurposing provides a systematic approach to discovering new therapy opportunities for bladder and prostate cancers, where heterogeneity and treatment resistance are still major challenges for treatment. The discovery of repurposed drugs that modulate dysregulated tumor-relevant pathways can be achieved by the comparison of drug and disease transcriptomic signatures. Signature reversal can help with tumor progression, therapy resistance, and cellular stress responses. Antiretrovirals have emerged as candidates for this process due to their effect on several cancer pathways.

It is, however, important to consider the challenges of translating transcriptomic predictions into clinical benefits presented by context-dependent gene expression, model-specific effects, and off-target mechanisms. Future progress in this area will require the integration of multi-omics, pharmacokinetic data, clinically relevant biomarkers, and functional validation together with transcriptomic data to translate results into clinical practice. Despite these challenges, and together with some improvements, gene expression-guided repurposing may support mechanism-informed repositioning of antiretrovirals in precision medicine for prostate and bladder cancers. We, therefore, frame antiretroviral repositioning as a mechanism-informed, biomarker-driven strategy, where transcriptomic context (subtype, lineage state, and pathway activity) defines the most plausible responder populations. This way, we distinguish antiretroviral repurposing from previous target and phenotypic-centered approaches by using transcriptomic and subtype strategies that are better aligned with precision oncology.

## Figures and Tables

**Table 1:** Antiretroviral repurposing for prostate cancer therapy.

Antiretroviral	Prostate Cancer Model	Key Molecular/Transcriptomic Effects	Phenotype Outcomes	Ref.
Ritonavir	PC-3, DU-145, DU-145DOC10, 22Rv1DOC8	- P-gp inhibition; - CYP3A4 inhibition; - NF-κB DNA binding activity inhibition	- Sensitization and reversal of chemoresistance to cabazitaxel and docetaxel - Growth inhibition - Apoptosis induction	[55,56]
Saquinavir	PC-3, DU-145, LNCaP	- 20S/26S proteasome activity inhibition - NF-κB activity inhibition	- Concentration-dependent apoptosis - Increased ionization radiation sensitivity - Reduced cell viability (alone and with 5-FU)	[57,58]
Efavirenz Etravirine	PC-3	- Time and concentration-dependent cytotoxicity	- Decreased cell viability - Impaired clonogenic growth/long-term proliferation	[59]
Efavirenz	In silico ligand-based virtual screening (BLAZE)	- Predicted PARP binding and inhibition	- Potential for DNA damage and apoptosis	[60]
Abacavir	PC-3, LNCaP	- LINE-1 upregulation - Chromatin remodeling, DNA replication, and protein modification gene expression are altered	- S-phase cell cycle arrest - Senescence - Impaired migration and invasion	[62]
Nelfinavir	PC-3, DU-145	- Broad RNA-seq transcriptomic changes in metabolism and stress response	- Castration-resistant cell proliferation inhibition	[63]
Nevirapine	Hormone-refractory prostate cell cancer	- Androgen receptor and disease progression gene signature reprogramming	- Restoration of androgen signaling	[64]

## Data Availability

No new data were created or analyzed in this study. Data sharing is not applicable to this article.
